# Focal dose escalation for prostate cancer using ^68^Ga-HBED-CC PSMA PET/CT and MRI: a planning study based on histology reference

**DOI:** 10.1186/s13014-018-1036-8

**Published:** 2018-05-02

**Authors:** Constantinos Zamboglou, Benedikt Thomann, Khodor Koubar, Peter Bronsert, Tobias Krauss, Hans C. Rischke, Ilias Sachpazidis, Vanessa Drendel, Nasr Salman, Kathrin Reichel, Cordula A. Jilg, Martin Werner, Philipp T. Meyer, Michael Bock, Dimos Baltas, Anca L. Grosu

**Affiliations:** 1 Department of Radiation Oncology, Medical Center – University of Freiburg, Faculty of Medicine, Robert-Koch Straße 3, 79106 Freiburg, Germany; 2 Division of Medical Physics, Department of Radiation Oncology, Medical Center – University of Freiburg, Faculty of Medicine, Freiburg, Germany; 3 Department of Pathology, Medical Center – University of Freiburg, Faculty of Medicine, Freiburg, Germany; 4 Department of Nuclear Medicine, Medical Center – University of Freiburg, Faculty of Medicine, Freiburg, Germany; 5 Department of Radiology, Medical Center – University of Freiburg, Faculty of Medicine, Freiburg, Germany; 6 Department of Urology, Medical Center – University of Freiburg, Faculty of Medicine, Freiburg, Germany; 7 Division of Medical Physics, Department of Radiology, Medical Center – University of Freiburg, Faculty of Medicine, Freiburg, Germany; 8 German Cancer Consortium (DKTK), Partner Site Freiburg, Freiburg, Germany; 9 Berta-Ottenstein-Programme, Faculty of Medicine, University of Freiburg, Freiburg, Germany

**Keywords:** Prostate cancer, Focal therapy, MRI, PSMA PET/CT

## Abstract

**Background:**

Focal radiation therapy has gained of interest in treatment of patients with primary prostate cancer (PCa). The question of how to define the intraprostatic boost volume is still open. Previous studies showed that multiparametric MRI (mpMRI) or PSMA PET alone could be used for boost volume definition. However, other studies proposed that the combined usage of both has the highest sensitivity in detection of intraprostatic lesions. The aim of this study was to demonstrate the feasibility and to evaluate the tumour control probability (TCP) and normal tissue complication probability (NTCP) of radiation therapy dose painting using ^68^Ga-HBED-CC PSMA PET/CT, mpMRI or the combination of both in primary PCa.

**Methods:**

Ten patients underwent PSMA PET/CT and mpMRI followed by prostatectomy. Three gross tumour volumes (GTVs) were created based on PET (GTV-PET), mpMRI (GTV-MRI) and the union of both (GTV-union). Two plans were generated for each GTV. Plan95 consisted of whole-prostate IMRT to 77 Gy in 35 fractions and a simultaneous boost to 95 Gy (Plan95^PET^/Plan95^MRI^/Plan95^union^). Plan80 consisted of whole-prostate IMRT to 76 Gy in 38 fractions and a simultaneous boost to 80 Gy (Plan80^PET^/Plan80^MRI^/Plan80^union^). TCPs were calculated for GTV-histo (TCP-histo), which was delineated based on PCa distribution in co-registered histology slices. NTCPs were assessed for bladder and rectum.

**Results:**

Dose constraints of published protocols were reached in every treatment plan. Mean TCP-histo were 99.7% (range: 97%–100%) and 75.5% (range: 33%–95%) for Plan95^union^ and Plan80^union^, respectively. Plan95^union^ had significantly higher TCP-histo values than Plan95^MRI^ (*p* = 0.008) and Plan95^PET^ (*p* = 0.008). Plan80^union^ had significantly higher TCP-histo values than Plan80^MRI^ (*p* = 0.012), but not than Plan80^PET^ (*p* = 0.472).

Plan95^MRI^ had significantly lower NTCP-rectum than Plan95^union^ (*p* = 0.012). No significant differences in NTCP-rectum and NTCP-bladder were observed for all other plans (*p* > 0.05).

**Conclusions:**

IMRT dose escalation on GTVs based on mpMRI, PSMA PET/CT and the combination of both was feasible. Boosting GTV-union resulted in significantly higher TCP-histo with no or minimal increase of NTCPs compared to the other plans.

**Electronic supplementary material:**

The online version of this article (10.1186/s13014-018-1036-8) contains supplementary material, which is available to authorized users.

## Background

Radiation therapy dose escalation for primary prostate cancer (PCa) can lower the risk of biochemical relapse [1]. Although toxicity from intensity modulated radiation therapy (IMRT) is manageable even at whole-prostate doses up to 86 Gy [2], recurrent PCa at the original tumour volume was still reported at this dose magnitude [3]. Therefore, further increase in dose escalation may be necessary to improve local tumour control [4]. In the last years, focal radiation therapy strategies evolved which limit normal tissue toxicity while enabling a further dose escalation to the tumour [5].

The exact delineation of the intraprostatic tumour mass is crucial for focal therapy strategies since the PCa volume should be covered by the imaging defined target region. Recently, two phase III trials (FLAME trial and HEIGHT trial) defined the intraprostatic boost volume by multiparametric MRI (mpMRI) [6]. However, first studies showed that PSMA PET/CT has a potential both in primary PCa detection and delineation [7, 8]. We examined the value of IMRT dose escalation on PSMA PET/CT-defined gross tumour volumes (GTVs) in a planning study. A boost of up to 95 Gy in 35 fractions resulted in significantly higher tumour control probability (TCP) values than a standard fractionation to the whole prostatic gland with 77 Gy in 35 fractions (96% vs. 70%). However, in 20% of the patients the dose escalation plans reached TCP values of around 80% [9].

In a comparison of PSMA PET and mpMRI for PCa detection, Eiber et al. [10] reported better area under the curve (AUC) values when PSMA PET and MRI information were combined, which we could confirm by performing a slice-by-slice comparison between mpMRI, PSMA PET/CT and histopathology after prostatectomy [11]. Sensitivities of 75%, 70% and 82% for PSMA PET, mpMRI and combined information were reported.

Furthermore, both studies pointed out that mpMRI and PSMA PET offer complementary information. However, there was a specificity of 67% for combined PSMA PET and mpMRI information [11], indicating that the combination may overestimate the true PCa amount within the prostate. Whether the increase in sensitivity and the decrease in specificity could be transferred to increased tumour control and normal tissue toxicity could not yet be answered.

The aim of this radiation therapy planning study was to demonstrate the technical feasibility of IMRT boosting based on GTVs derived from PSMA PET/CT, mpMRI or combined (PSMA PET and mpMRI) information in patients with primary PCa. Additionally, we compared the value of mpMRI, PSMA PET/CT and their combination for IMRT dose escalation guidance by calculating the TCPs based on the dose distribution in PCa within co-registered histology. The strength of this planning study is that the TCP calculation is based on the histological data, while the radiation treatment planning is done based on multimodal imaging derived GTVs. The normal tissue complication probabilities (NTCPs) for bladder and rectum were calculated.

## Methods

### Patients

The study cohort consisted of 10 patients with primary PCa (intermediate and high risk according to NCCN-guidelines) who had PSMA PET/CT and mpMRI scans prior to radical prostatectomy. Their characteristics are described in Additional file 1: Table S1. Written informed consent was obtained from each patient, and the institutional review board approved this study.

### PET/CT imaging

PET/CT scans using the ligand ^68^Ga-HBED-CC-PSMA [12] were either performed with a 64-slice GEMINI TF PET/CT or a 16-slice GEMINI TF BIG BORE PET/CT (both Philips Healthcare. USA). A detailed description of our ^68^Ga-HBED-CC-PSMA PET/CT imaging protocol is given in our previous publication [13]. To ensure the comparability of the quantitative measurements, both imaging systems were cross-calibrated. Patients underwent the whole-body PET scan starting 1 h after injection. The uptake of ^68^Ga-PSMA-HBED-CC was quantified by standardized uptake values (SUV).

### MR imaging

MR images were acquired either on a 3 Tesla system (Trio Tim, Siemens, Germany / 7 patients) or on a 1.5 Tesla system (Aera and Avanto, Siemens, Germany / 3 patients). All systems were equipped with a surface phased array (Body Matrix) in combination with an integrated spine array coil. No endo-rectal coil was used. Essentially, T2-weighted fast spin echo (T2W-TSE) images, diffusion weighted images (DWI) and dynamic contrast-enhanced (DCE) perfusion images were acquired. A detailed description of the MR imaging protocol is given in [13].

### Image co-registration

**Fig. 1:**
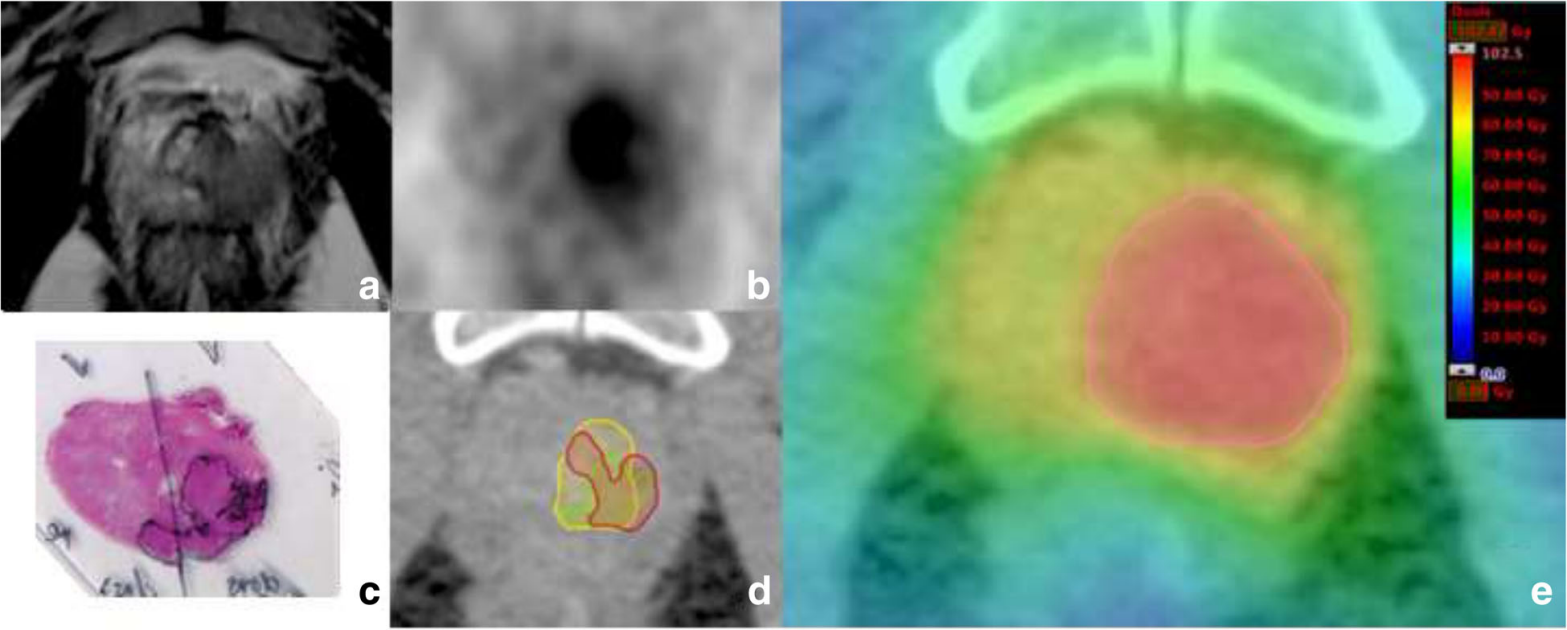
Transverse T2-weighted image (**a**) shows a hypointense signal in the left lobe. (**b**) shows a PSMA PET image with intense focal uptake located in the left lobe. Haematoxylin and eosin gross section histopathology shows a large tumour focus in the left lobe (**c**). (**d**) shows a transverse CT image (from PSMA PET/CT scan) with projected GTVs (green: GTV-histo, yellow: GTV-PET, red: GTV-MRI) for patient 9. In (**e**) the colourwash representation for Plan^95union^ is presented. The PTV of the boost volume is marked in red

After formalin-fixation, the resected prostate was placed in a special holder and a CT scan was performed. Subsequently, whole-mount step sections were cut using an in-house cutting device and processed by a board-certified pathologist. According to our previous study [11], histopathological information was digitalized to create GTV-histo and registered on in-vivo CT (PSMA PET/CT scans), taking into account the non-linear shrinkage and distortion of the resected prostate tissue (Fig. 1). Subsequently, in-vivo PET/CT datasets (including GTV-histo) were imported into iPlan (iPLAN RT image 4.1, BrainLAB. Germany). Axial TSE-, DWI- ADC maps and DCE-MRI images were matched with in-vivo CT images using mutual information registration. If visual assessment showed an anatomical mismatch, a manual adjustment was performed based on anatomical markers. For alignment between PET and CT images the pre-set registration was used. Thus, CT/PET/MRI and histopathology data were registered in the same reference frame.

### Generation of contours

Contours of the GTVs were generated in iPLAN. Based on our recent results, GTV-PET was created semi-automatically using a threshold of 30% of SUVmax within the prostate [7]. Two board-certified radiologists delineated GTV-MRI in consensus using T2W, DWI and DCE-sequences to characterize each lesion. Lesions with visually determined PI-RADs v2 [14] score 4 or higher were included in the analysis. With respect to PI-RADs v2 criteria, T2W-TSE and DWI images were primarily used for delineation of transition zone and peripheral zone lesions, respectively. The addition of GTV-PET and GTV-MRI was classified as GTV-union. Subsequently, the in-vivo CT including all above described GTVs was transferred to the RT planning system Eclipse v13.5 (Varian, USA) and contours for the prostate, seminal vesicles, and surrounding Organs at risk were generated. Clinical target volume 1 (CTV1) was defined as the prostate and the seminal vesicles. CTV2 was defined as the prostate and half of the seminal vesicles (high risk patients) or the basis of the seminal vesicles (intermediate risk patients). CTV1, CTV2, GTV-MRI, GTV-PET and GTV-union were enlarged by an isotropic margin of 4 mm to create the respective PTVs.

### IMRT planning

Rapid Arc IMRT treatment plans were created in Eclipse v13.5 (Varian, USA). For each patient two different focal radiation therapy regiments were simulated. A moderate dose escalation was planned according to Pinkawa et al. [15] and a more intense dose escalation was planned in analogy to the experimental arm of the Flame trial [6]. The simultaneous integrated boost was delivered based on PET (PTV-PET), MRI (PTV-MRI) or combined PSMA PET and mpMRI information (PTV-union).

#### 1.) FLAME trial protocol

To simulate the experimental arm of the FLAME trial we planned 52.8 Gy in 24 fractions on PTV1 and 24.2 Gy in 11 fractions on PTV2 (EQD2_α/β=3Gy_ = 80 Gy) with a concomitant boost to PTV-MRI (Plan95^MRI^), PTV-PET (Plan95^PET^), PTV-union (Plan95^union^) with a dose of 95 Gy in 35 fractions (EQD2_α/β=3Gy_ = 109 Gy). Dose constraints for bladder and rectum were taken from the FLAME protocol [6].

#### 2.) Pinkawa et al. protocol

Treatment planning was performed according to [15]. We planned 54 Gy in 27 fractions on PTV1 and 22 Gy in 11 fractions on PTV2 (EQD2_α/β=3Gy_ = 76 Gy) with a simultaneous dose escalation to PTV-MRI (Plan80^MRI^), PTV-PET (Plan80^PET^), PTV-union (Plan80^union^) with a dose of 80 Gy in 38 fractions (EQD2_α/β=3Gy_ = 80 Gy). Dose constraints for bladder and rectum were taken from the study protocol [15]. In case of an overlap between the boost volumes and the rectal wall a maximum dose to the rectum of up to 80 Gy was defined as a minor deviation.

During planning, dose constraints for the organs at risk had the highest priority. In order to achieve comparable plans for the different boost volumes the dose distribution within the corresponding PTVs was optimized to be as homogeneous as possible (see Additional file 2: Table S2a and 2b).

### Radiobiological treatment plan evaluation

The TCP and NTCP calculations were performed using the research version of BIOTOP/BIOSPOT (Pi-medical, Greece) and MATLAB R2017a (The MathWorks, USA). The summation of 3D dose distributions, EQD2 as well as TCP and NTCP calculations were performed at voxel level. For TCP calculations, a radiobiological model based on the linear quadratic (LQ) Poisson model [16–20] was used. TCP calculations were performed based on GTV-histo (TCP-histo), assuming it to represent the true clinical response.

For the TCP calculations, we used the parameter α/β = 1.93 [21] and the tumor cell density ρ = 2.8 × 10^8^ cells /cm^3^ for intermediate and high-risk patients [22–24]. The value for α (α = 0.1335 Gy^− 1^) was chosen in order to achieve an average TCP-histo value of 70% over all patients for the standard arm fractionation of the FLAME trial (77 Gy in 35 fractions) [6]. For a detailed description of the TCP calculation methodology performed in this study, please see Additional file 3 and our previous publication [9].

To calculate NTCPs of non-uniform dose distributions the relative seriality model was used [18, 25–27]. The following parameters were selected for bladder and rectum according to [28]. For bladder D50 = 80 Gy (symptomatic contracture and volume loss. EQD2), s = 1.3 and γ = 2.59 and for rectum D50 = 80 Gy (severe proctitis/necrosis/stenosis/fistula. EQD2), s = 0.75 and γ = 1.79 were chosen. The γ-values were calculated based on the listed k-values [9]. For both organs, an α/β ratio of 3 Gy was assumed according to a recent study [29].

### Statistical analysis

Statistical analyses were performed with Prism 7 (GraphPad, USA) and Microsoft Excel 2010 (Microsoft, USA). The Wilcoxon matched pairs signed-rank test was used with a threshold for statistical significance of < 0.05.

## Results

GTV-histo, GTV-PET, GTV-MRI and GTV-union in average amounted to 15 ± 12%, 17 ± 13%, 10 ± 9% and 20 ± 14% of the total intraprostatic volume (mean 54.17 ± 24.35 ml), respectively (Table 1).

**Table 1:** GTV volumes for each patient

	% of prostatic volume				
Patient	GTV-Histo	GTV-PET	GTV-MRI	GTV-union	Volume prostate (ml)
1	17%	39%	8%	41%	31.9
2	10%	23%	8%	24%	31.4
3	32%	25%	25%	36%	61.8
4	25%	9%	19%	22%	53.6
5	2%	2%	1%	2%	110.2
6	3%	4%	3%	5%	48.7
7	2%	3%	1%	4%	70
8	4%	10%	4%	11%	60
9	19%	24%	22%	33%	26.5
10	33%	26%	10%	26%	47.6
Mean	15%	17%	10%	20%	54.2
SD ±	12%	13%	9%	14%	24.4

GTV-histo was not significantly smaller than GTV-union (*p* = 0.1) and GTV-PET (*p* = 0.715) but significant larger than GTV-MRI (p = 0.047) in Wilcoxon matched pairs signed-rank test. Mean prostatic volume (delineated in CT) was 54.2 ± 24.4 ml

**Fig. 2:**
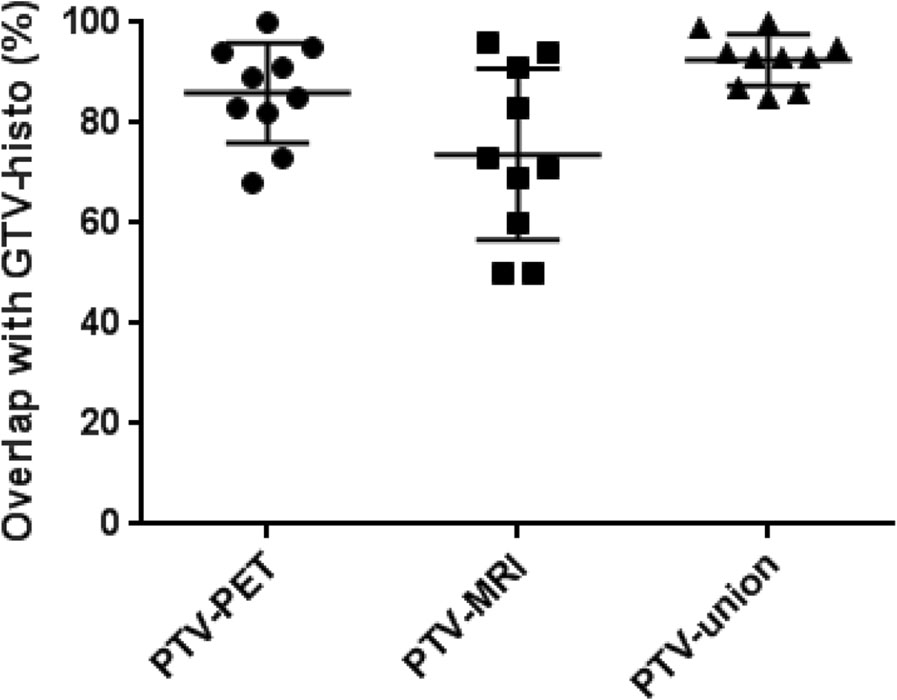
The middle horizontal bars represent the mean values and the upper and lower bars the standard deviations. In Wilcoxon matched pairs signed-rank test, GTV-histo overlapped significantly higher with PTV-union than with PTV-PET (*p* = 0.016) and PTV-MRI (*p* = 0.002), respectively

In average, 86 ± 10%, 74 ± 17% and 93 ± 5% of GTV-histo overlapped with PTV-PET, PTV-MRI and PTV-union, respectively (Fig. 2).

For all patients the target volume objectives as well as the OAR dose constraints were met. For Plan95^PET^, Plan95^MRI^ and Plan95^union^ the mean doses for GTV-histo were 95.3 ± 2.6 Gy, 93.3 ± 2.6 Gy and 96.3 ± 1.5 Gy, respectively. For Plan80^PET^, Plan80^MRI^ and Plan80^union^ the mean doses for GTV-histo were 80.7 ± 0.4 Gy, 79.9 ± 0.8 Gy and 80.8 ± 0.5 Gy, respectively. Additional file 4: Figure S1 shows dose volume histograms (DVHs) for GTV-histo, averaged for all plans and all patients.

TCP-histo values are listed in Table 2.

**Table 2:** TCP-histo values

	Plan95^PET^	Plan95^MRI^	Plan95^union^	Plan80^PET^	Plan80^MRI^	Plan80^union^
Mean (%)	94.7	96.9	99.7	73.0	70.8	75.5
Maximum (%)	100.0	100.0	100.0	94.0	94.0	95.2
Minimum (%)	69.6	86.4	97.4	25.1	30.2	33.0

Mean, maximum and minimum TCP-histo values over all patients for all plans are listed

**Fig. 3:**
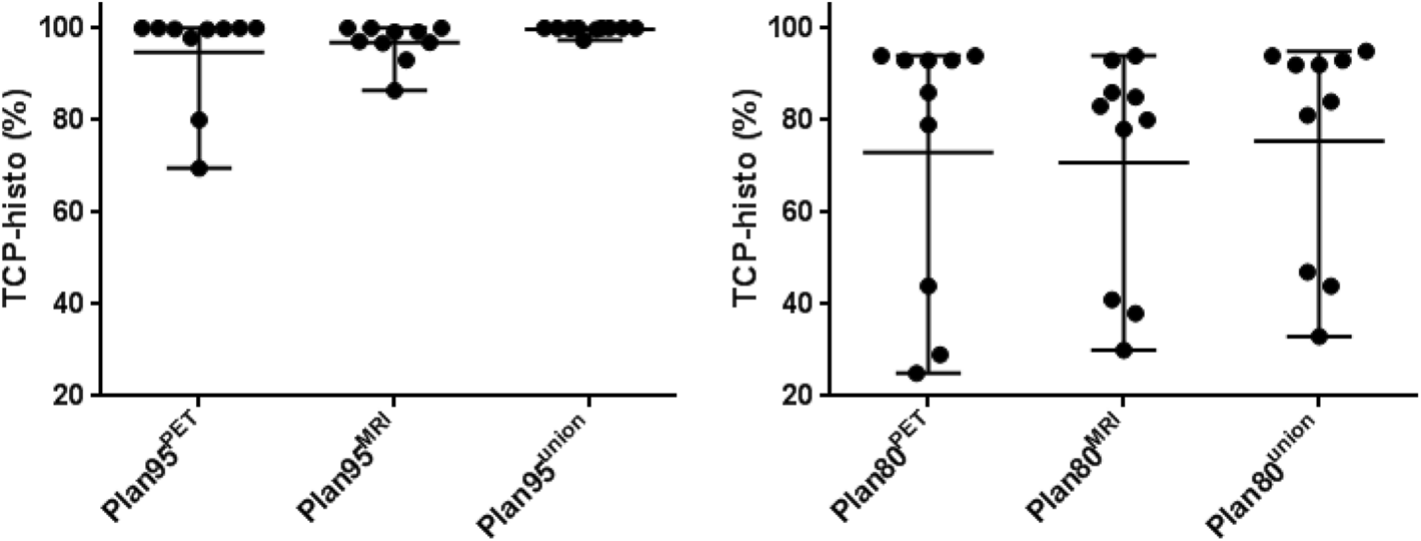
The middle horizontal bars represent the mean values and the upper and lower bars the respective maximum and minimum values. Wilcoxon matched pairs signed-rank test showed that Plan95^union^ had significantly higher TCP values than both Plan95^MRI^ and Plan95^PET^, respectively (*p* < 0.05). Plan80^union^ only had significantly higher TCP values than Plan80^MRI^ (*p* < 0.05) but not than Plan80^PET^ (*p* = 0.5). There were no significant differences in TCP-histo values between Plan80/95^MRI^ and Plan80/95^PET^ (*p* = 0.371 for Plan80 and *p* = 0.844 for Plan95)

Plan95^union^ had significantly higher TCP-histo values than Plan95^MRI^ (*p* = 0.008) and Plan95^PET^ (*p* = 0.008). Plan80^union^ had significantly higher TCP-histo values than Plan80^MRI^ (*p* = 0.012). There were no significant differences in TCP-histo values between Plan80^PET^ and Plan80^union^ (*p* = 0.472). Whether the dose escalation was delivered based on PET or mpMRI information had no impact on TCP-histo values for both protocols (*p* > 0.05, Fig. 3).

**Fig. 4:**
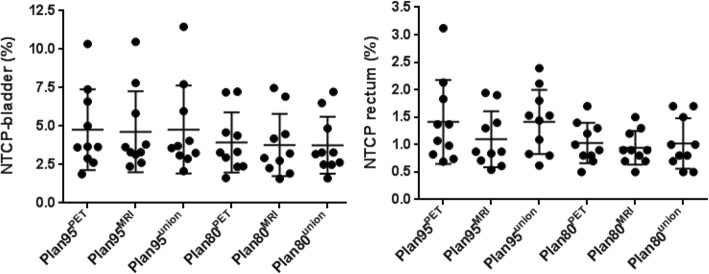
For all patients NTCP values for bladder and rectum were presented for all plans. The middle bars represent the mean values and the upper and lower bars the standard deviations. Wilcoxon matched pairs signed-rank test showed that no significant differences in NTCP values for the different Plans when dose was delivered in analogy to the Pinkawa protocol (*p* > 0.05). When dose was delivered in analogy to the Flame trial a significant reduction in NTCP-rectum values was observed for Plan95^MRI^ compared to Plan95^union^ (*p* = 0.012) and Plan95^PET^ (*p* = 0.047). There were no significant differences in NTCP-bladder values for Plan95 (*p* > 0.05)

NTCP calculations for bladder and rectum revealed no significant differences for all plans (*p* > 0.05, Fig. 4), with the exception that Plan95^MRI^ had significantly lower NTCP-rectum values than Plan95^union^ (*p* = 0.012) and Plan95^PET^ (*p* = 0.047), respectively.

## Discussion

A reliable delineation of the intraprostatic tumor burden is a prerequisite for implementation of focal therapy approaches in treatment of primary PCa. Most of the published studies used mpMRI to define the target for focal therapy guidance [5]. Our group [7] and others [8, 30] illustrated a great potential for PSMA PET/CT based delineation of primary PCa. However, two recent studies examined the role of combined PSMA PET and mpMRI information for primary PCa localization based on histology reference. Both reported higher sensitivities when the combined information was used compared to PSMA PET or mpMRI alone [10, 11]. Accordingly, we could show in this study that GTV-histo overlapped significantly higher with PTV-union, which was generated based on combined mpMRI and PSMA PET information, than with PTV-PET or PTV-MRI alone. Furthermore, mean GTV-union was slightly larger than mean GTV-PET (*p* > 0.05) and mean GTV-histo (*p* > 0.05) and it was significantly larger than GTV-MRI (*p* < 0.05). The main questions for this study were whether a focal dose escalation, which is guided by combined PSMA PET and mpMRI information, is technically feasible and if an increase in TCP values is achieved compared to boosting GTVs based on PSMA PET or mpMRI alone. We performed TCP-histo calculations based on registered histological information after prostatectomy, which should correlate with the real PCa distribution and should also predict the true clinical outcome.

This study confirmed the technical feasibility for prescription doses and dose constraints of the FLAME trial [6] and Pinkawa et al. [15]. These two clinical protocols were chosen since they applied different prescription doses for the prostate (EQD2_α/β=3Gy_ = 76 Gy [15] and 80 Gy [6]) and the boost volume (EQD2_α/β=3Gy_ = 80 Gy [15] and 109 Gy [6]) using similar fractions. NTCP values for rectum and bladder were identical for all plans, except of a slight decrease in NTCP-rectum values for Plan95^MRI^ (mean NTCP-rectum was 1.09 for Plan95^MRI^, 1.41 for Plan95^PET^ and 1.42 for Plan95^union^).

TCP-histo values were significantly higher for Plan95^union^ and Plan80^union^ compared to the plans in which the boost volume was derived from mpMRI or PSMA PET/CT alone. This observation can most likely be ascribed to the high overlap between PTV-union and GTV-histo. In average 86%, 74% and 93% of GTV-histo overlapped with PTV-MRI, PTV-PET and PTV-union, respectively. For Plan80, the assumed correlation between GTV-histo coverage and resulting TCP-histo was confirmed as the mean TCP-histo was indeed higher for Plan80^PET^ than it was for Plan80^MRI^. Interestingly though, for Plan95^MRI^ the mean TCP-histo was higher than for Plan95^PET^. A good coverage of the main PCa mass by PTV-MRI serves as an explanation for this observation. Since the FLAME protocol deliveres a higher dose to the entire prostate than the Pinkawa protocol (difference of EQD2_α/β=3Gy_ = 4 Gy), missing small PCa lesions with the boost volume has a lower impact on the TCP for the FLAME protocol than it has for the Pinkawa protocol. For ultra-focal therapy approaches like high intensity focused ultrasound (HIFU) [31], or focal low−/high-dose rate brachytherapy [32, 33] the treatment is focused solely within the imaging defined target or region. Thus, a high coverage of the PCa mass may be even more crucial than for the two IMRT protocols which were used in this study.

As expected, TCP-histo values for Plan95 had a much lower range than TCP-histo values for Plan80^union^ (Fig. 3 and Table 2), indicating that the intensity of dose escalation has a higher impact than the modality which was chosen for boost-volume delineation. Dose escalation up to 95 Gy on PTV-PET and PTV-MRI alone reached excellent results (TCP-histo > 95%) in 8 of 10 patients. Furthermore, only a small difference in mean TCP-histo values between Plan80^PET^ and Plan80^union^ was measured (73% vs. 76). This might be seen as an indicator that a single imaging modality (PSMA PET or mpMRI) is sufficient for GTV-delineation, particularly when considering the overutilization of diagnostic imaging in current health systems [34]. However, several studies showed that PSMA PET and mpMRI offer complementary information in detection of primary PCa [10, 11, 13]. 22% [11] to 32% [10] of prostatic areas were classified as positive by one modality and negative by the other. Furthermore, we found very little to no differences in NTCP values for bladder and rectum between the plans. Future studies are needed to characterize those patient populations (e.g. by Gleason score or PSA serum levels) in which a combined usage of PSMA PET and mpMRI is necessary and to differentiate them from the remaining majority of cases where only a single imaging modality is sufficient. Until this question is finally answered, the combined usage of PET and mpMRI for GTV-delineation ensures the best therapeutic ratio.

Beyond GTV-delineation for dose escalation guidance, the combined usage of mpMRI and PSMA PET/CT offers further advantages in the clinical workflow of patients with primary PCa. MRI provides a better soft tissue contrast than CT images and is likewise superior for prostatic gland delineation [35]. On the other hand, PSMA PET/CT is superior in lymph node [36] and skeletal [37] staging compared to conventional imaging, indicating that PSMA PET/CT may also be used as a “one-stop shop staging” modality for patients with intermediate and high-risk PCa.

An important issue of this study is the uncertainty in registration of PET/CT, mpMRI and histopathology (e.g. non-linear shrinkage of the prostate after prostatectomy or different rectum and bladder fillings during imaging) [38]. The usage of hybrid PET/MRI systems [10] might account for the registration uncertainties between the PET and mpMRI, but these systems are currently not widely available. A second issue of this study is the margin used for PTV generation since the PTV affects the NTCP (dose to rectum and bladder) as well as the TCP (potential shifts of GTV-histo out of the dose escalation area). The FLAME trial (5–8 mm) [39] and the Pinkawa protocol (3–8 mm) [15] used larger PTV margins around the prostate than our study. On the other hand, the Pinkawa protocol [15] applied a margin of 3–4 mm to create the intraprostatic dose escalation volume and the FLAME trial [39] used no margin for this at all. In the current study we expanded both the prostate and the intraprostatic GTVs with an isotropic margin of 4 mm to create the respective PTVs. At our department the patients with primary PCa receive daily fiducial marker-based position verification to account for interfractional movements. Additionally, an adaptive radiotherapy [40] protocol based on repeated cone-beam CT scans was established in order to calculate the average position of the targets and the organs at risk. Therefore, at our department the PTV mainly accounts for the intrafractional movement during IMRT (maximum movement of 2 mm in > 85% of datasets after 6 min of RT [41]) and possible registration errors between CT and MRI information (approx. 2 mm [42]). The usage of 4 mm margins around the prostate in our study could be considered as a possible reason for keeping the dose constraints for rectum and bladder. However, a planning study by Lips et al. [43] simulated an intraprostatic dose escalation and analyzed the effect of different margins (2–8 mm) around the prostate on the dose distributions for bladder and rectum. The authors observed that the dose constraints for both organs were met for all margins. Our group simulated intrafractional movement during PSMA PET guided simultaneous integrated boost IMRT for patients with primary PCa [44]. By using the same PTV margins as the current study we showed that intrafractional movement in average does not have any significant effect on the TCP and can even increase the TCP if the boost volume is surrounded by a sufficiently high dose plateau.

Another potential limitation of this study is that the clinically derived parameters of the biological model used in this study have not been validated through prospective clinical trials. To account for this issue a previous planning study used 15 different parameter value combinations for TCP calculations. The observed variance between the TCP-histo values for the different parameter value sets was low [9], which justifies the approach in this study.

In summary, we could show in 10 patients that the concept of a focal dose escalation is feasible on GTVs delineated by combined PSMA PET and mpMRI information. High TCPs were achieved with acceptable NTCPs. These findings need to be further validated in a prospective dose escalation trial for patients with primary PCa.

## Conclusion

In patients with primary PCa IMRT dose escalation is feasible using GTVs defined on multimodal image data (mpMRI and PSMA PET/CT). It achieves significantly higher TCP-histo values with minimal to no increase of NTCP values compared to IMRT dose escalation on GTVs derived solely based on one imaging modality.

## Additional files

Additional file 1: **Table S1.** Patient characteristics. (PDF 106 kb)

Additional file 2: **Tables S2a + b.** 1. FLAME protocol / 2. Pinkawa protocol. Dose characteristics after IMRT planning based on different protocols (PDF 82 kb)

Additional file 3: A. Additional information on TCP calculation / B. Additional information on NTCP calculation. (PDF 278 kb)

Additional file 4: **Figure S1.** Dose volume histograms (DVHs) for GTV-histo, averaged for all plans and all patients. (PDF 169 kb)

## Abbreviations

approx: Approximately
AUC: Area under the curve
CT: Computer tomography
CTV1: Clinical target volume
DCE: Dynamic contrast-enhanced images
DWI: Diffusion weighted images
EQD2: Equivalent dose in 2 Gy per fraction
GTV: Gross tumour volume
Gy: Gray
IMRT: Intensity modulated radiation therapy
MRI: Magnetic resonance imaging
NTCP: Normal tissue complication probability
PCa: Prostate cancer
PET: Positron emission tomography
PTV: Planning target volume
SUV: Standardized uptake value
T2W-TSE: T2-weighted fast spin echo images
TCP: Tumour control probability
