# Supplementary material for: Focal dose escalation for prostate cancer using 68Ga-HBED-CC PSMA PET/CT and MRI: a planning study based on histology reference
Source: Radiat Oncol. 2018 May 2;13:81. doi: 10.1186/s13014-018-1036-8 (PMC5930745; doi:10.1186/s13014-018-1036-8)
Supplement: Supplementary file 1 — Table S1. Patient characteristics. (PDF 106 kb) [file 13014_2018_1036_MOESM1_ESM.pdf]

Patient	Age (y)	PSA (ng/ml)	TNM	Gleason score
1	67	6.1	pT3a pN1 cM0	3+4 (7a)
2	61	10.6	pT2c pN0 cM0	3+4 (7a)
3	52	51.1	pT3b pN1 cM0	5+4 (9)
4	60	49.0	pT2c pN1 cM0	3+4 (7a)
5	73	25.5	pT2c pN0 cM0	3+4 (7a)
6	59	9.2	pT2c pN0 cM0	4+3 (7b)
7	74	8.8	pT2c pN0 cM0	3+4 (7a)
8	74	15.3	pT2c pN0 cM0	3+4 (7a)
9	51	17.4	pT3a pN0 cM0	4+3 (7b)
10	48	23.2	pT3b pN1 cM0	4+3 (7b)
Mean	61.4	21.5		
SD ±	9.4	16.8		

Table S1. Patient characteristics
