# Supplementary material for: Focal dose escalation for prostate cancer using 68Ga-HBED-CC PSMA PET/CT and MRI: a planning study based on histology reference
Source: Radiat Oncol. 2018 May 2;13:81. doi: 10.1186/s13014-018-1036-8 (PMC5930745; doi:10.1186/s13014-018-1036-8)
Supplement: Supplementary file 2 — Tables S2a + b. 1. FLAME protocol / 2. Pinkawa protocol. Dose characteristics after IMRT planning based on different protocols (PDF 82 kb) [file 13014_2018_1036_MOESM2_ESM.pdf]

1. FLAME protocol

Patient	Dose	**PTV-PET**	**PTV-MR**	**PTV-Union**
**1**	*Dmin (Gy)*	73.01	80.87	72.52
	D98% (Gy)	81.17	89.63	79.32
	D95% (Gy)	85.46	90.60	84.17
	D50% (Gy)	94.74	97.10	94.48
	D2% (Gy)	98.00	100.83	98.96
	Dmax (Gy)	100.02	104.25	101.46
	**Dmean (Gy)**	**93.25**	**96.33**	**93.10**
**2**	*Dmin (Gy)*	71.58	82.27	59.96
	D98% (Gy)	82.67	87.92	72.84
	D95% (Gy)	86.15	88.81	78.62
	D50% (Gy)	95.94	94.66	94.75
	D2% (Gy)	99.90	98.44	99.61
	Dmax (Gy)	101.51	101.41	102.13
	**Dmean (Gy)**	**94.69**	**94.05**	**92.90**
**3**	*Dmin (Gy)*	73.17	66.70	78.83
	D98% (Gy)	83.89	80.21	79.68
	D95% (Gy)	85.96	84.72	84.76
	D50% (Gy)	95.19	94.73	95.06
	D2% (Gy)	98.26	97.58	98.09
	Dmax (Gy)	99.98	100.07	100.09
	**Dmean (Gy)**	**93.64**	**93.10**	**93.46**
**4**	*Dmin (Gy)*	84.24	81.64	77.64
	D98% (Gy)	88.65	86.33	85.97
	D95% (Gy)	89.44	87.97	86.90
	D50% (Gy)	94.17	94.89	92.90
	D2% (Gy)	98.22	97.80	97.91
	Dmax (Gy)	100.05	100.28	99.79
	**Dmean (Gy)**	**93.88**	**93.94**	**92.61**
**5**	*Dmin (Gy)*	88.01	87.52	88.48
	D98% (Gy)	90.85	90.18	91.03
	D95% (Gy)	91.66	90.85	91.74
	D50% (Gy)	96.17	95.28	96.25
	D2% (Gy)	100.05	98.20	99.92
	Dmax (Gy)	102.48	99.17	101.30
	**Dmean (Gy)**	**95.91**	**94.88**	**95.97**
**6**	*Dmin (Gy)*	80.94	82.56	80.22
	D98% (Gy)	88.21	89.62	86.71
	D95% (Gy)	89.90	90.43	89.33
	D50% (Gy)	95.89	94.53	96.19
	D2% (Gy)	100.82	97.75	99.67
	Dmax (Gy)	103.61	100.72	101.76
	**Dmean (Gy)**	**95.53**	**94.17**	**95.38**
**7**	*Dmin (Gy)*	77.51	86.41	80.29
	D98% (Gy)	88.31	89.68	88.39

	D95% (Gy)	89.83	90.48	89.58
	D50% (Gy)	95.33	95.46	95.98
	D2% (Gy)	98.97	99.11	99.85
	Dmax (Gy)	102.95	100.90	102.23
	**Dmean (Gy)**	**94.79**	**95.02**	**95.33**
**8**	Dmin (Gy)	83.65	86.99	83.66
	D98% (Gy)	89.38	90.26	89.55
	D95% (Gy)	90.39	90.92	90.51
	D50% (Gy)	96.75	96.02	97.25
	D2% (Gy)	101.40	100.40	102.13
	Dmax (Gy)	104.46	103.75	105.45
	**Dmean (Gy)**	**96.19**	**95.70**	**96.62**
**9**	Dmin (Gy)	78.88	82.13	78.61
	D98% (Gy)	86.03	87.07	85.56
	D95% (Gy)	87.34	88.25	87.14
	D50% (Gy)	95.50	95.54	95.78
	D2% (Gy)	99.56	99.66	99.69
	Dmax (Gy)	102.07	101.19	102.05
	**Dmean (Gy)**	**94.37**	**94.82**	**94.59**
**10**	Dmin (Gy)	74.49	82.70	72.78
	D98% (Gy)	83.07	87.30	82.57
	D95% (Gy)	85.21	88.38	85.82
	D50% (Gy)	93.58	96.17	95.76
	D2% (Gy)	101.29	99.47	99.20
	Dmax (Gy)	102.94	101.34	101.32
	**Dmean (Gy)**	**93.47**	**95.12**	**94.25**

2. Pinkawa protocol

Patient	Dose	**PTV-PET**	**PTV-MR**	**PTV-Union**
**1**	Dmin (Gy)	71.96	74.06	71.88
	D98% (Gy)	75.02	77.57	75.87
	D95% (Gy)	76.40	77.94	77.27
	D50% (Gy)	79.84	79.63	80.19
	D2% (Gy)	82.55	81.93	82.56
	Dmax (Gy)	84.17	84.56	83.96
	**Dmean (Gy)**	**79.71**	**79.66**	**80.06**
**2**	Dmin (Gy)	72.98	73.90	72.20
	D98% (Gy)	75.39	78.22	75.78
	D95% (Gy)	77.64	78.55	77.90
	D50% (Gy)	80.80	80.25	80.61
	D2% (Gy)	83.16	82.88	83.17
	Dmax (Gy)	84.44	84.64	84.76
	**Dmean (Gy)**	**80.58**	**80.35**	**80.50**
**3**	Dmin (Gy)	73.53	70.27	70.59
	D98% (Gy)	76.71	75.05	74.43

	D95% (Gy)	78.00	76.95	76.80
	D50% (Gy)	80.48	80.08	80.32
	D2% (Gy)	82.78	82.45	82.81
	Dmax (Gy)	84.07	84.91	84.58
	**Dmean (Gy)**	**80.32**	**79.90**	**80.05**
**4**	*Dmin (Gy)*	74.66	70.65	73.09
	D98% (Gy)	78,72	76.65	77.62
	D95% (Gy)	79.20	77.44	78.27
	D50% (Gy)	81.00	80.22	80.31
	D2% (Gy)	83.90	83.40	82.74
	Dmax (Gy)	85.97	84.99	84.19
	**Dmean (Gy)**	**81.07**	**80.17**	**80.25**
**5**	*Dmin (Gy)*	76.59	76.58	77.37
	D98% (Gy)	78.37	78.17	78.53
	D95% (Gy)	78.62	78.45	78.77
	D50% (Gy)	80.22	80.12	80.14
	D2% (Gy)	82,61	82.85	82.72
	Dmax (Gy)	83.91	83.79	83.20
	**Dmean (Gy)**	**80.32**	**80.27**	**80.36**
**6**	*Dmin (Gy)*	73.53	73.48	72.84
	D98% (Gy)	73.51	75.97	74.96
	D95% (Gy)	75.85	77.40	76.44
	D50% (Gy)	80.07	79.73	79.91
	D2% (Gy)	83.10	82.52	82.61
	Dmax (Gy)	84.72	84.01	84.42
	**Dmean (Gy)**	**79.77**	**79.83**	**79.77**
**7**	*Dmin (Gy)*	72.60	76.93	72.33
	D98% (Gy)	77.48	78.21	77.71
	D95% (Gy)	78.25	78.45	78.81
	D50% (Gy)	79.89	79.94	80.10
	D2% (Gy)	82.85	82.15	82.87
	Dmax (Gy)	84.54	82.93	83.94
	**Dmean (Gy)**	**79.99**	**80.03**	**80.14**
**8**	*Dmin (Gy)*	72.18	74.73	72.17
	D98% (Gy)	77.36	77.77	77.29
	D95% (Gy)	78.15	78.24	78.05
	D50% (Gy)	80.14	80.09	80.37
	D2% (Gy)	83.13	82.95	83.18
	Dmax (Gy)	84.75	84.39	84.56
	**Dmean (Gy)**	**80.28**	**80.20**	**80.31**
**9**	*Dmin (Gy)*	72.97	73.65	72.73
	D98% (Gy)	75.91	76.82	75.81
	D95% (Gy)	77.24	77.81	77.26
	D50% (Gy)	80.01	80.18	80.14
	D2% (Gy)	83.18	83.05	83.28
	Dmax (Gy)	85.90	85.82	85.01
	**Dmean (Gy)**	**79.98**	**80.18**	**80.05**

**10**	*Dmin (Gy)*	72.59	75.78	71.69
	D98% (Gy)	75.89	78.23	76.32
	D95% (Gy)	77.74	78.68	77.84
	D50% (Gy)	80.75	80.51	80.5
	D2% (Gy)	83.92	82.87	83.16
	Dmax (Gy)	86.92	83.98	85.51
	**Dmean (Gy)**	**80.63**	**80.52**	**80.37**
