# Supplementary material for: Focal dose escalation for prostate cancer using 68Ga-HBED-CC PSMA PET/CT and MRI: a planning study based on histology reference
Source: Radiat Oncol. 2018 May 2;13:81. doi: 10.1186/s13014-018-1036-8 (PMC5930745; doi:10.1186/s13014-018-1036-8)
Supplement: Supplementary file 3 — A. Additional information on TCP calculation / B. Additional information on NTCP calculation. (PDF 278 kb) [file 13014_2018_1036_MOESM3_ESM.pdf]

A. Additional information on TCP calculation

GTV was considered to be the histopathology proven and defined cancer volume in the prostate. TCP was calculated according to the Poisson distribution as [17-21]:

$$TCP = P_B = P_{GTV}(\{D\}, V) = \prod_{i=1}^{M} e^{-\rho_{cell}\Delta V_i P(D_i)}$$

With P(D_i) the survival probability for the dose D_i [18,19]:

$$P(D_i) = e^{-(\frac{EQD2_i}{D50})(e\gamma - ln ln 2)} \quad \text{or equivalently} \quad P(D_i) = e^{-\alpha\, EQD2_i\, (1+\frac{2Gy}{\alpha/\beta})}$$

M was the total number of voxels within GTV or of bins of the differential dose volume histogram for total dose in the GTV.ρ_{cell} represented the cancer cell density in GTV and ΔV_i was the voxel volume or the total volume corresponding to the ith dose bin. D50 was the equieffective dose for 2Gy per fraction resulting to 50% tumor control probability and γ was the maximum normalized dose-response gradient [19,26]. $EQD2_i$ was the equieffective dose for 2Gy per fraction for the total dose D_i for the ith -voxel or i^{th}-dose bin [9,21]:

$$EQD2_i = D_i(1 + \frac{D_i/N}{\alpha/\beta})/(1 + \frac{2Gy}{\alpha/\beta})$$

With N the total number of fractions for delivering the total dose D_i. α/β values described the curvature of the cell survival curve described by the linear-quadratic model, where α described the slope of the initial linear part of the curve.

B. Additional information on NTCP calculation

NTCP from non-uniform dose distributions were calculated using the relative seriality model [19,26-28]:

$$P_I = 1 - \prod_{j=1}^{N_{organs}} \left(1 - P_I^j\right) = 1 - \prod_{j=1}^{N_{organs}} \left(1 - [1 - \prod_{i=1}^{M_j} (1 - P^j(D_i)^{s_j})^{\Delta v_i}]^{1/s_j}\right)$$

With P(D_i) the complication probability rate for the dose D_i and was given by the same formula as for TCP, where here D50 is the equieffective dose for 2Gy per fraction resulting to 50% complication probability. Δv_i was the volume fraction being irradiated to dose D_i and s was the parameter which expresses the degree of seriality (the value varies from *s* close to zero for nearly parallel organs and upwards for increasing seriality).

The maximum normalized dose-response gradient γ in the survival probability function was be calculated from the parameter k of the corresponding logit probability distribution according to the following:

$k = 4\,\gamma_{50}$ and $\gamma_{50} = \frac{ln2}{2}(e\gamma - lnln2)$, thus $\gamma = \frac{\frac{k}{2ln2} + lnln2}{e}$

with γ_{50} the slope of the dose-response curve at the 50% response level.
