# Supplementary figures and images for: Focal dose escalation for prostate cancer using 68Ga-HBED-CC PSMA PET/CT and MRI: a planning study based on histology reference
Source: Radiat Oncol. 2018 May 2;13:81. doi: 10.1186/s13014-018-1036-8 (PMC5930745; doi:10.1186/s13014-018-1036-8)

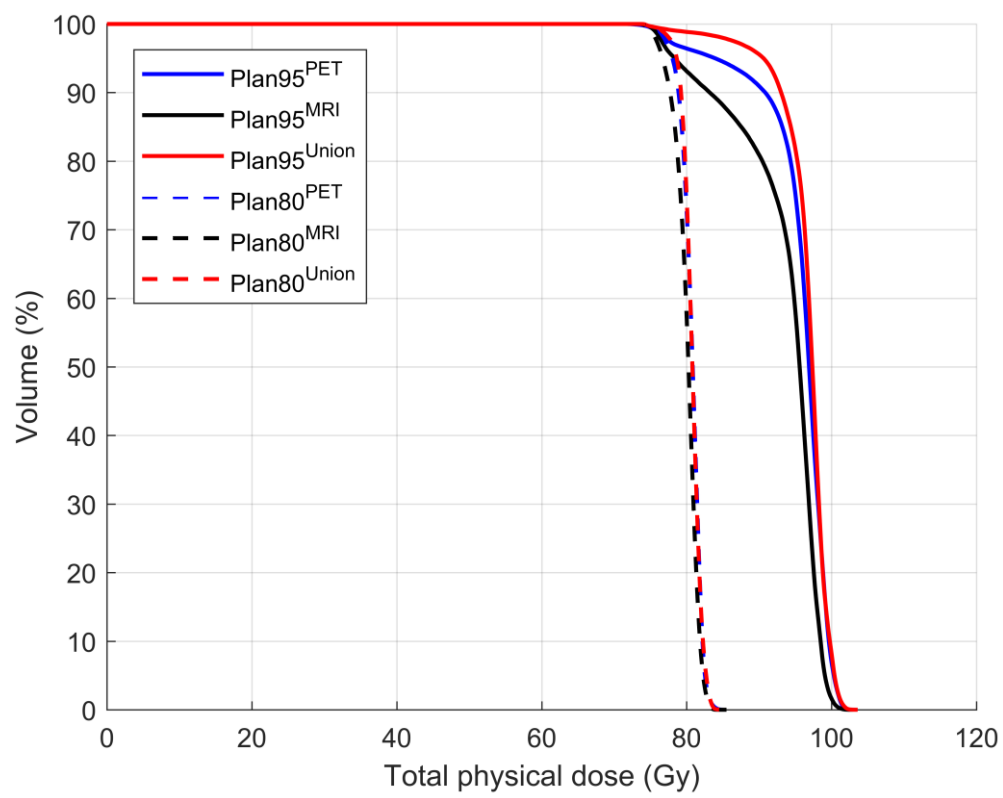

Supplement: Supplementary file 4 — Figure S1. Dose volume histograms (DVHs) for GTV-histo, averaged for all plans and all patients. (PDF 169 kb) [file 13014_2018_1036_MOESM4_ESM.pdf]
